# Collagen Modulates the Biological Characteristics of WJ-MSCs in Basal and Osteoinduced Conditions

**DOI:** 10.1155/2022/2116367

**Published:** 2022-08-28

**Authors:** Vun Vun Hiew, Peik Lin Teoh

**Affiliations:** Biotechnology Research Institute, Universiti Malaysia Sabah, Jalan UMS, 88400 Kota Kinabalu, Sabah, Malaysia

## Abstract

Transcriptomic analysis revealed mesenchymal stem/stromal cells (MSCs) from various origins exhibited distinct gene and protein expression profiles dictating their biological properties. Although collagen type 1 (COL) has been widely studied in bone marrow MSCs, its role in regulating cell fate of Wharton jelly- (WJ-) MSCs is not well understood. In this study, we investigated the effects of collagen on the characteristics of WJ-MSCs associated with proliferation, surface markers, adhesion, migration, self-renewal, and differentiation capabilities through gene expression studies. The isolated WJ-MSCs expressed positive surface markers but not negative markers. Gene expression profiles showed that COL not only maintained the pluripotency, self-renewal, and immunophenotype of WJ-MSCs but also primed cells toward lineage differentiations by upregulating *BMP2* and *TGFB1* genes. Upon osteoinduction, WJ-MSC-COL underwent osteogenesis by switching on the transcription of *BMP6/7* and *TGFB3* followed by activation of downstream target genes such as *INS*, *IGF1*, *RUNX2*, and *VEGFR2* through p38 signalling. This molecular event was also accompanied by hypomethylation at the *OCT4* promoter and increase of H3K9 acetylation. In conclusion, COL provides a conducive cellular environment in priming WJ-MSCs that undergo a lineage specification upon receiving an appropriate signal from extrinsic factor. These findings would contribute to better control of fate determination of MSCs for therapeutic applications related to bone disease.

## 1. Introduction

Mesenchymal stromal/stem cells (MSCs) with self-renewal and multiple differentiation potentials are a promising source for tissue engineering and regenerative medicine. Besides having an excellent *ex vivo* expansion capability, they can be obtained from any part of the human body [1]. Bone marrow (BM) has been the main source and widely studied MSCs since its first discovery. However, due to invasive procurement of BM-MSCs, Wharton jelly (WJ) emerges as a promising source owing to its high cell yield, multipotent differentiation potentials, distinct immunomodulatory properties, easily attainable, and less ethical concern [2]. Besides that, the differentiation and proliferation capabilities of BM-MSCs are age-dependent [3]. Although BM-MSCs showed higher osteogenic potential, they also evoked immune responses. Thus, WJ-MSCs with superior immunomodulatory properties become an invaluable source for allogenic therapy [4].

Many studies have demonstrated that MSCs from various origins or even the same tissue show variable and distinct gene or protein expression profiles even though they display similar morphology, leading to heterogeneity in their biological properties [5–7]. In comparison to BM-MSCs, inferiority of WJ-MSCs in osteogenic and adipogenic lineages was attributed by aberrant gene expression involved in WNT pathways [5]. Besides that, the transcriptome profile of WJ-MSCs is enriched with genes associated with adhesion, proliferation, and immunomodulatory and neurotrophic factor when compared to other adult MSCs [4, 8, 9].

To sustain the pertinence of MSCs in clinical settings, biomaterials have been employed for promoting cell growth and guiding or tailoring MSC behaviors toward specific applications. Collagen type 1 (COL) is a natural component of the extracellular matrix (ECM) found in animal tissues. Due to its biocompatibility, biodegradability, and low immunogenicity, COL alone or its derivatives have been widely used in tissue engineering. Mounting evidence demonstrated that collagen interacted with cell surface integrins to modulate cell behaviors such as cell attachment, proliferation, and differentiation [10].

Previous studies have shown that COL promoted adhesion, proliferation, and osteogenic differentiation of BM-MSCs [11]. Mochizuki et al. [12] demonstrated that COL promoted cell growth of human dental pulp stem cells cultured in xenogeneic serum-free media and sustained the survival and proliferative potential of overconfluent cells without affecting stem cell properties and chromosomal stability. COL was also found to induce osteogenic differentiation of amniotic membrane- (AM-) MSCs at basal condition but without significant change in cell growth [13]. Additionally, WJ-MSCs embedded onto the COL scaffold were shown to promote chondrogenesis and cartilage repair in the knee [14]. The expression of cartilage specific markers *Sox9*, *Col2a1*, and *Acan* was upregulated in BM-MSCs encapsulated onto COL hydrogel [15]. Despite tremendous studies, modulating effects of collagen and the underlying mechanisms are not fully understood especially in other MSCs as current knowledge is mainly based on the studies on BM-MSCs.

In this study, we studied the effect of collagen on the biological characteristics of WJ-MSCs including adhesion, migration, proliferation, immunomodulation, surface markers, self-renewal, and lineage-differentiation capabilities through gene expression studies. Then, we examined whether collagen modulates these changes by influencing DNA methylation and histone acetylation. Finally, we summarized the discrepancies of collagen in directing osteogenic cell fate under basal and induced conditions.

## 2. Materials and Methods

### 2.1. Ethic Permission and Sample Collection

This study was ethically approved by the Ethics and Research Committee of Universiti Malaysia Sabah with approval code: JKEtika 1/16 (1). Human placentas were collected from healthy full terms through caesarean delivery after informed consents were obtained. Placenta samples were obtained from Damai Specialist Hospital and KPJ Hospital, Kota Kinabalu, and isolation was processed within 24 hours.

### 2.2. Isolation, Culture, and Passaging of WJ-MSCs

Firstly, the umbilical cord (UC) was removed from the placenta. WJ-MSCs were isolated from the UC matrix using an enzymatic digestion method. The UC was washed with Dulbecco's phosphate-buffered saline (DPBS) (Gibco-Invitrogen, Carlsbad, CA, USA) for three times to remove blood cells. The UC was sectioned into a few pieces about 4-5 cm in length. After removing blood vessels and amniotic epithelium, the remaining tissue was WJ. WJ was chopped and minced, then transferred to 50 mL falcon tubes containing 0.1% type 1 collagenase (Worthington, Minnesota, USA) for 2 hours of digestion with constant agitation at 180 rpm at 37°C. After centrifugation, cell pellet was resuspended in DMEM/F12 media containing 10% fetal bovine serum, 1% GlutaMAX, 1% antibiotic-antimycotic, and 1% ascorbic acid (Merck, Darmstadt, Germany) then transferred into a T-25 flask and incubated at 37°C. Adherent cells were proceeded to passaging using 0.25% trypsin-EDTA until cells reached about 80% confluency. Cell culture media and reagents were obtained from Gibco-Invitrogen (Carlsbad, CA, USA) if not specifically mentioned. WJ-MSCs were also cultured in tissue culture plate or microplate-coated collagen type 1 (#152034: 6-well plates, #152036: 96-well plates) purchased from Thermo Fisher Scientific (Waltham, MA, USA).

### 2.3. Cell Morphology and Proliferation

Cell proliferation was examined using PrestoBlue Viability Reagent (Thermo Fisher Scientific, Waltham, MA, USA) following the manufacturer's protocol. WJ-MSCs of density at 5 × 10^3^ cells/well were seeded in 96-well plates for overnight. After incubation, 10 *μ*L of prewarmed PrestoBlue reagent was added then incubated at 37°C for 1 hour in the dark. Fluorescent readings were obtained using Infinite 200 microplate reader (Tecan, USA). The measurement was repeated every two days until cell detachment. The cell morphology at various passages was also examined under an inverted microscope (Olympus IX73, Japan).

### 2.4. Differentiation Assays

WJ-MSCs were plated at a density of 5 × 10^3^ in 96-well plates coated with or without collagen. After reaching 80-90% confluency, culture media was replaced with either StemPro osteogenesis, adipogenesis, or chondrogenesis differentiation media (Gibco-Invitrogen, Carlsbad, CA, USA) and incubated for 14 days. Medium was changed at least twice per week. WJ-MSCs were at first fixed with 10% formaldehyde (Merck, Darmstadt, Germany) in DPBS for 30 minutes at room temperature. Osteogenic, adipogenic, and chondrogenic cells were stained with 2% Alizarin Red (ScienCell, Carlsbad, CA, USA), Oil Red O (Merck, Darmstadt, Germany), and Alcian Blue solution (Sigma-Aldrich, St. Louis, MO), respectively. Staining was performed according to the manufacturers' protocols. After washing with DPBS, cells were observed under an inverted microscopy.

### 2.5. Immunocytochemistry

Cells were seeded at 1 × 10^4^ density on round coverslips overnight. After washing, fixation with 4% paraformaldehyde (Thermo Fisher Scientific, Waltham, MA, USA) was performed for 15 minutes; then cells were rinsed with DPBS for 3 times. Blocking was performed using 1% bovine serum albumin (Sigma-Aldrich, St Louis, Missouri, USA) for 30 minutes then washed with DPBS. After incubation with primary antibody for 1 hour at room temperature, cells were washed again. The secondary antibody was added for another 1 hour. Antibodies used in this study were CD90-conjugated FITC, CD14-conjugated AF594, and CD34-conjugated AF488. After washing, cells were mounted with DAPI mounting medium (Gibco, Life Technologies, USA).

### 2.6. RNA Extraction and RT-PCR

Total RNA was extracted using TransZol Up Plus RNA Kit (Transgen Biotech, Beijing, China) following the manufacturer's procedures. RT-PCR was performed using TransScript One-Step RT-PCR SuperMix (Transgen Biotech, Beijing, China) according to manufacturer's protocol. First-strand cDNA synthesis was performed at 45°C for 30 minutes, 94°C for 2 minutes, followed by 35 cycles of 94°C for 30 seconds, 52-55°C for 30 seconds, 72°C for 1 minute, and then final extension at 72°C for 5 minutes. Primers targeting stemness and differentiation genes are listed in Table 1, while primers targeting surface markers were based on sequences published by Ali et al. [16]. Band intensity was quantified using BioRad Image Lab software. Relative expression was obtained after normalization with *ACTB* expression levels then compared to the control.

### 2.7. PCR Array

The cDNA synthesis was performed using RT^2^ First Strand Kit (Qiagen, Maryland, USA) following the manufacturer's protocol. A 20 *μ*L reaction mixture containing RT^2^ SYBR Green ROX Fast Mastermix (Qiagen, Maryland, USA), cDNA, and RNase-free water were loaded into each well of Human Mesenchymal Stem Cell RT^2^ Profiler PCR Array (PAHS-082, SABiosciences, Qiagen). PCR program was set according to the manufacturer's protocol. Samples with *C*_*T*_ value ≥ 33 was considered a negative call. The fold change values were measured using data from real time cycler with ΔΔ*C*_*T*_ method and analysed through the web-based software RT^2^ Profiler PCR Array Data Analysis (Qiagen, Maryland, USA). Fold regulation values > 1 and <-1 indicate upregulation and downregulation, respectively.

### 2.8. Protein Extraction and Western Blotting

Total protein was extracted using Total Protein Extraction kit (Merck Millipore, Darmstadt, Germany) following the manufacturer's protocol. Protein lysates were separated using 10% SDS-PAGE. After electrophoresis, protein was transferred using BioRad Trans-blot Turbo Transfer System for 50 minutes at 15 V. After transferring, the blot was washed with Tris-buffered saline containing Tween-20 (TBST) and incubated for 1 hour in a blocking buffer consisting of 5% bovine serum albumin (Sigma-Aldrich, St Louis, Missouri, United States). The blot was probed with primary antibodies at 4°C with overnight agitation, followed by 1-hour incubation with HRP-conjugated antibody. Band intensity was analysed using Image Lab Software (BioRad Laboratories, California, United States). Histone H3 and H3K9Ac were purchased from Abcam (Cambridge, United Kingdom), while phospho-ERK1/2, phospho-p38, and p38 were obtained from BioLegend (San Diego, California, United States).

### 2.9. Bisulphite Sequencing

DNA was extracted using QIAmp DNA Mini Kit (Qiagen, Maryland, USA) according to the manufacturer's protocol. Bisulphite conversion was performed using EZ DNA Methylation-Direct Kit (Zymo Research, Irvine, California). The conversion was carried out according to the manufacturer's protocol. The bisulphite primers listed in Table 2 were designed using MethPrimer software version 2.0. For each reaction, 2 *μ*L of converted DNA was used. PCR amplification was carried out using GoTaq DNA polymerase (Promega, Wisconsin, USA). The initial denaturation was set at 95°C for 2 minutes, followed by 35 cycles of denaturation at 95°C for 40 seconds, annealing at 55°C for 40 seconds, extension at 72°C for 60 seconds, and then final extension at 72°C for 5 minutes. PCR product was separated on 2% agarose gel; desired band was cut then purified using QIAquick Gel Extraction Kit (Qiagen, Maryland, USA) following the manufacturer's instructions. Purified amplicon from two independent biological samples was successfully sequenced.

### 2.10. Statistical Analysis

All experiments were carried out using three independent biological replicates which were expressed in mean ± SEM. Statistical analysis was done using paired *t*-test and two-way ANOVA. *p* value < 0.05 was considered statistically significant.

## 3. Results

### 3.1. Cell Morphology and Proliferation

**Figure 1:**
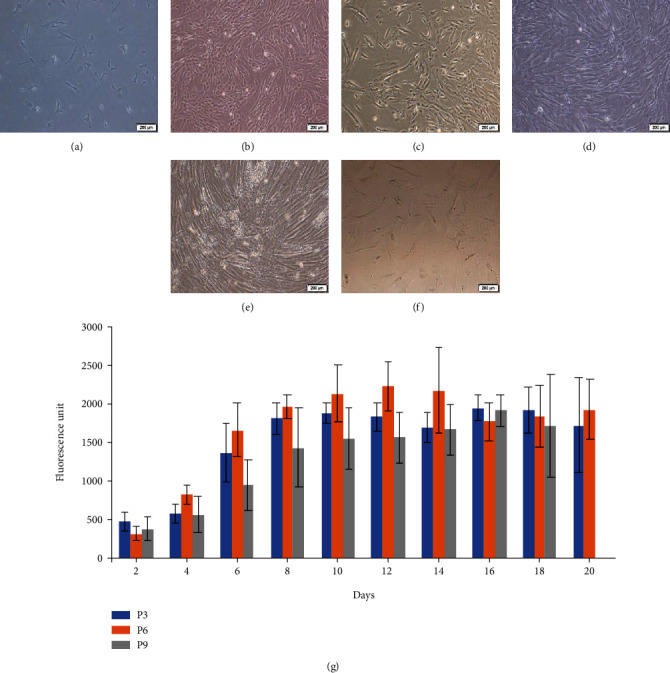
Morphological observation and proliferation of WJ-MSCs. (a) P0, (b) P1, (c) P3, (d) P6, (e) P9, and (f) P11. Scale bar = 200 *μ*m. (g) Cells at P3, P6, and P9 were cultured for up to 20 days. Data obtained from three biological independent samples.

Cells from different passages were microscopically observed as depicted in Figure 1. At passage 0 to 6 (P0-P6), the isolated cells were attached with spindle-shaped morphology (Figures 1(a)–1(d)). However, the morphology at P9 became flattened, stretched, and longer in length (Figure 1(e)). At P11, cells were enlarged in shape and a sharp decrease in cell number was observed (Figure 1(f)). To ensure sufficient cells for subsequent analysis, a cell passage that gave the highest proliferative capacity was determined. Among these passages, cells at P6 proliferated faster and produced relatively higher cell numbers than P3 and P9 (Figure 1(g)).

### 3.2. The Expression of Surface Markers and Differentiation Potentials

**Figure 2:**
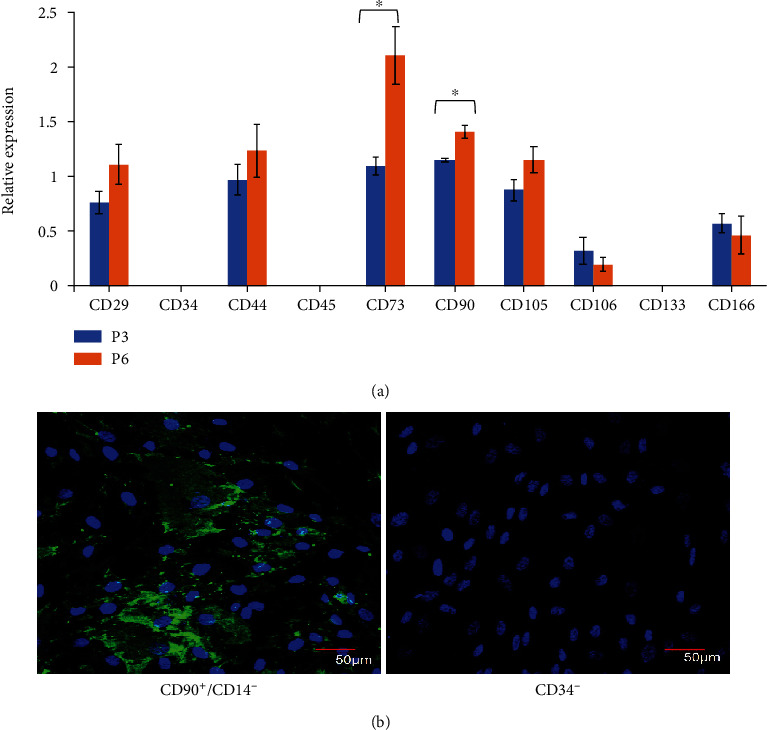
The mRNA and protein expression of surface markers in WJ-MSC. (a) The gene expression of positive surface markers was quantified after relative to *ACTB*. Data obtained from three independent biological samples. Asterisk (∗) indicates statistical significance (*p* < 0.5). (b) Immunocytochemical staining was performed against CD90 (green), CD14 (red), and CD34 (red). The cells were counter-stained using DAPI (blue).

To verify the identity and multipotent of the isolated cells, surface markers commonly expressed in MSCs were assessed. The results showed that they expressed positive surface markers including *CD73*, *CD90*, *CD105*, *CD29*, *CD44*, *CD166*, and *CD106* in both P3 and P6. But negative markers such as *CD34*, *CD45*, *CD14*, and *CD133* were undetectable. A significant increase in *CD73* and *CD90* expression was also noticeable at P6 (Figure 2(a)). The protein expression of CD90, CD14, and CD34 is depicted in Figure 2(b). See Figure S1 for immunophenotyping of the isolated WJ-MSCs.

**Figure 3:**
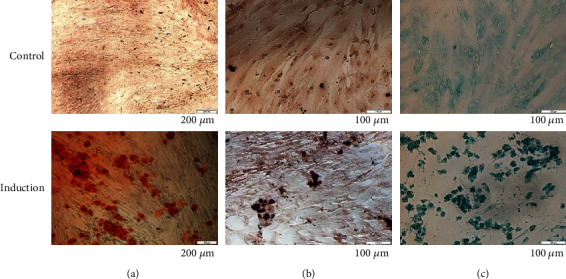
Trilineage differentiation potentials of WJ-MSCs. Cells cultured in basal and induction media were stained with (a) Alizarin red, (b) Oil red O, and (c) Alcian blue.

Alizarin red staining showed calcium deposition, which is a characteristic of osteoblast formation after osteogenic induction (Figure 3(a)). When the induced cells were stained with Oil red O and Alcian blue, lipid and glycosaminoglycan accumulation were formed in the differentiated cells (Figures 3(b) and 3(c)), suggesting the occurrence of adipogenesis and chondrogenesis, respectively.

### 3.3. The Effects of Collagen on Cell Morphology, Proliferation, and Lineage Differentiation

**Figure 4:**
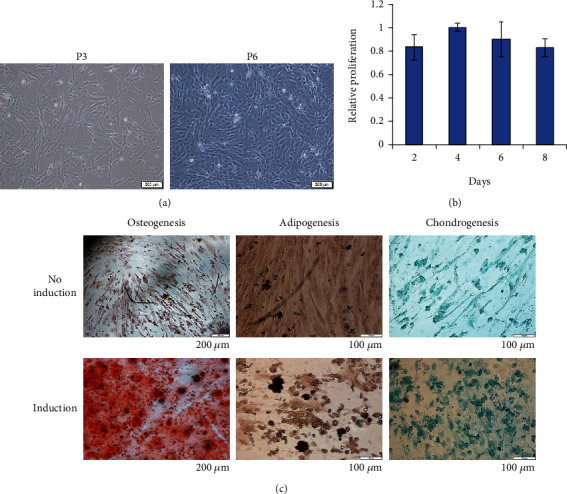
The effects of collagen on the morphology, proliferation, and lineage differentiation of WJ-MSCs. (a) Cell morphological observation at P3 and P6 on a collagen-coated plate. (b) Relative proliferation of cells cultured on collagen plate compared to the control. Data obtained from three independent biological samples. (c) Staining of differentiated cells cultured on the collagen surface with or without induction.

Cells cultured on collagen- (COL-) coated plates remained spindle-shaped at both P3 and P6 (Figure 4(a)). Although collagen had been shown to promote MSC proliferation, our results demonstrated comparable proliferative potential (Figure 4(b)) as reported by Akhir and Teoh [13]. We further explored the effect of collagen on lineage commitments of WJ-MSCs. Like previously reported, WJ-MSCs were differentiated into osteocytes, adipocytes, and chondrocytes upon stimulation with the respective induction media (Figure 4(c); bottom panel). Under basal condition, little formation of calcium, lipid, and glycosaminoglycan was also observed in WJ-MSC-COL (Figure 4(c); upper panel).

### 3.4. The Effects of Collagen in Surface Marker Expressions

**Figure 5:**
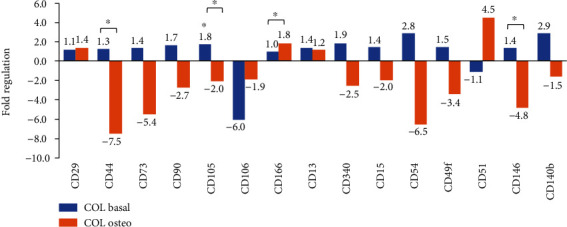
The effects of collagen on the gene expression of surface markers. WJ-MSC-COL cultured in basal media was compared to WJ-MSCs without COL, while WJ-MSC-COL cultured in osteogenic media was compared to WJ-MSC-COL in basal media. All data were obtained from three independent biological samples. Asterisk (∗) indicates statistical significance (*p* < 0.05).

We then examined the effect of collagen on the expression of surface markers. COL scaffold has increased the mRNA expression of some MSC-specific markers ≥1.5-fold, including *CD105* (*ENG*), *CD90* (*THY1*), *CD54* (*ICAM1*), *CD340* (*ERBB2*), *CD49f* (*ITGA6*), and *CD140b* (*PDGFRB*). In contrast, the *CD106* expression was reduced 6-fold (Figure 5; blue bars). However, most surface marker expressions were downregulated upon osteogenic induction (Figure 5; orange bars). The significant affected genes were *CD44*, *CD105* (*ENG*), and *CD146* (*MCAM*), while only *CD166* (*ALCAM*) and *CD51* (*ITGAV*) were upregulated.

### 3.5. Collagen Modulates the Expression of Differentiation Genes

**Figure 6:**
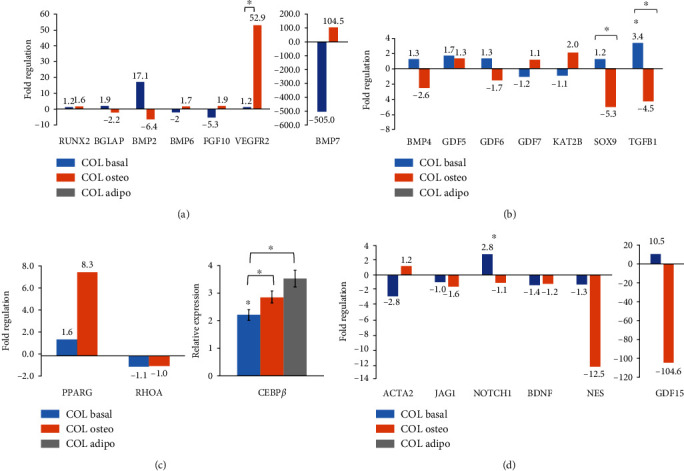
Collagen modulated the expression of differentiation genes. The expression of (a) osteogenic, (b) chondrogenic, (c) adipogenic, and (d) other lineage differentiation genes. WJ-MSC-COL cultured in basal media was compared to WJ-MSCs without COL, while WJ-MSC-COL cultured in osteogenic media was compared to WJ-MSC-COL in basal media. Asterisk (∗) indicates statistical significance (*p* < 0.05). The *CEBPβ* expression was obtained through semiquantitative RT-PCR. All data were obtained from three independent biological samples.

We next studied how collagen regulates lineage differentiation genes including those involved in osteogenesis, chondrogenesis, adipogenesis, tenogenesis, myogenesis, and neurogenesis. At basal condition, we observed upregulation of *BGLAP/OCN* and *BMP2* expressions (Figure 6(a); blue bars). Upon osteogenic stimulation, the downregulation of *BMP2* was reciprocally followed by the upregulation of *BMP6* and *BMP7*, suggesting a switching role of BMP family members in inducing osteogenesis by osteogenic media which subsequently increased the expression of downstream target genes such as *RUNX2, FGF10*, and *VEGFR2* (Figure 6(a); orange bars).

An increase in the expression of *TGFB1*, an upstream regulator of chondrogenic pathway, and its downstream target, *GDF5*, was noticeable in WJ-MSC-COL under uninduced condition (Figure 6(b); blue bars). In contrast, the expression of TGF*β*/BMP signalling pathway was concomitantly suppressed upon osteogenic induction. This was also supported by significant downregulation of *SOX9* and *TGFB1* (Figure 6(b); orange bars).

The role of collagen in promoting adipogenesis of WJ-MSCs without adipogenic stimulation could be evidenced by the upregulation of the two key regulators, *PPARG* and *CEBPβ*. Surprisingly, osteogenic induction did not seem to inhibit their expressions as a greater increase was observed (Figure 6(c)). However, the *CEBPβ* expression was elevated more in WJ-MSC-COL stimulated with adipogenic media (Figure 6; grey bar).

Furthermore, the expression of key tenogenic genes including *BMP2*, *GDF15*, and *TGFB1* at basal condition was profoundly increased in 17.1-fold, 10.5-fold, and 3.4-fold, respectively (Figures 6(a), 6(b)– 6(d); blue bars), suggesting that COL could promote tenogenesis. Except for *NOTCH1* which was upregulated significantly, other genes involved in myogenesis (*ACTA2*, *JAG1*) and neurogenesis (*BDNF*, *NES*) were unaltered or downregulated in WJ-MSC-COL (Figure 6(d); blue bars). Although NOTCH1 promoted BM-MSCs differentiation to cardiomyocyte [17], its inhibition had reduced proliferation but promoted osteogenesis [18]. Nonetheless, the activation of NOTCH1 signalling suppressed the initiation of osteogenesis but is essential during terminal differentiation and mineralization in osteocytes [19].

### 3.6. Collagen Modulates the Expression of Stemness, Immunomodulatory, and Other MSC Genes

**Figure 7:**
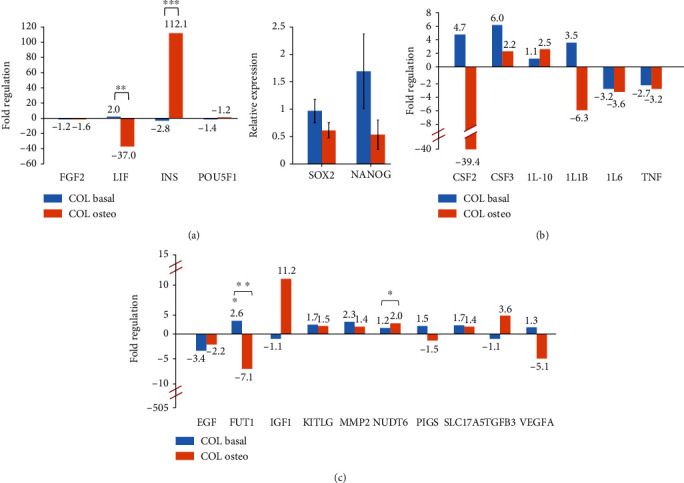
Collagen modulated the expression of (a) stemness, (b) immunomodulatory, and (c) other MSC genes. WJ-MSC-COL cultured in basal media was compared to WJ-MSCs without COL, while WJ-MSC-COL cultured in osteogenic media was compared to WJ-MSC-COL in basal media. Asterisk (∗) indicates statistical significance (*p* < 0.05). The *SOX2* and *NANOG* expressions were obtained through semiquantitative RT-PCR. All data obtained from three independent biological samples.

Without induction, the *LIF* expression was upregulated while the *INS* expression was downregulated (Figure 7(a); blue bars). Upon induction, osteogenesis caused a significant inverse effect on their expression (Figure 7(a); orange bars). Semiquantitative RT-PCR results showed that COL maintained *SOX2* and *NANOG* expression in noninduced condition, but their expression was reduced after osteogenesis (Figure 7(a)). The downregulation of *LIF*, *SOX2*, and *NANOG* in WJ-MSC-COL was in accordance with the occurrence of osteogenesis.

The presence of collagen elevated the expression of *CSF2*, *CSF3*, *IL10*, and *IL1B*, but suppressed *IL6* and *TNF* (Figure 7(b); blue bars). Other upregulated MSC genes were *FUT1*, *KITLG* (*SCF*), *MMP2*, *PIGS*, and *SLC17A5* (Figure 7(c); blue bars), indicating collagen potentially promoted migration and adhesion. After osteoinduction, the expression of proinflammatory genes was decreased except *CSF3* and *IL10* remained upregulated (Figure 7(b); orange bars). Besides that, *FUT1*, *PIGS*, and *VEGFA* expressions were obviously reduced, while the expressions of *IGF1*, *TGFB3*, and *NUDT6* were upregulated (Figure 7(c); orange bars). Downregulation of *EGF* and unchanged expression of *FGF* and *IGF1* suggested collagen did not enhance the proliferation of WJ-MSCs at basal condition (Figure 7; blue bars).

### 3.7. Collagen Modulates Gene Expression via DNA Methylation, Histone Acetylation, and MAPK Signalling

**Figure 8:**
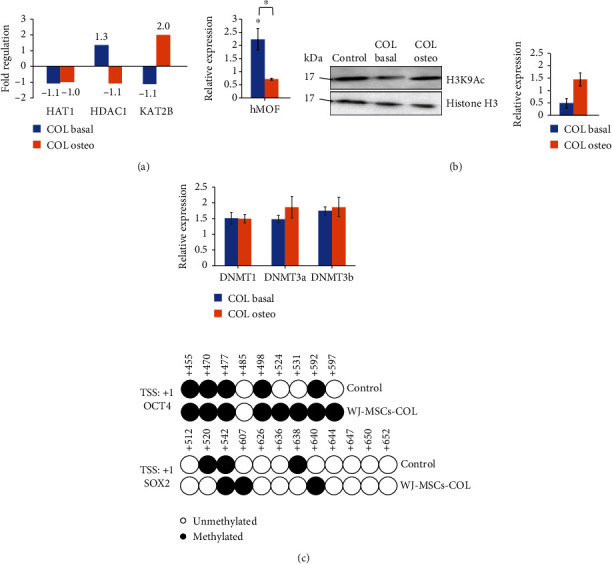
Collagen influenced the gene expression of epigenetic regulators. Cells were cultured on collagen scaffold at basal or osteogenic condition. (a) The gene expression of histone acetyltransferases. (b) Representative Western blot was probed with antibodies as indicated. Relative protein expression level obtained after normalization using histone H3. (c) Top: the gene expression of DNA methyltransferases; bottom: the methylation status at the promoter regions of *OCT4* and *SOX2*. Circles represent the CpG sites. All data were obtained from three independent biological samples. Asterisk (∗) indicates significance (*p* < 0.05).

COL influenced the expression of epigenetic regulators by upregulating *hMOF* and *DNMTs* (Figures 8(a) and 8(c); blue bars). Upon osteoinduction, hypermethylation was noticeable at the promoter of *OCT4*, while the methylation status at *SOX2* promoter was unchanged (Figure 8(c); bottom panel). In contrast, H3K9 acetylation was suppressed initially but upregulated after osteogenic induction which is substantiated by an increase in histone acetyltransferase, *KAT2B* (Figure 8(a); orange bars). The expression of *hMOF* was increased in WJ-MSC-COL but decreased upon osteogenic induction (Figure 8(a)). However, the upregulated expression of *DNMT*s was preserved upon osteoinduction (Figure 8(c)).

**Figure 9:**
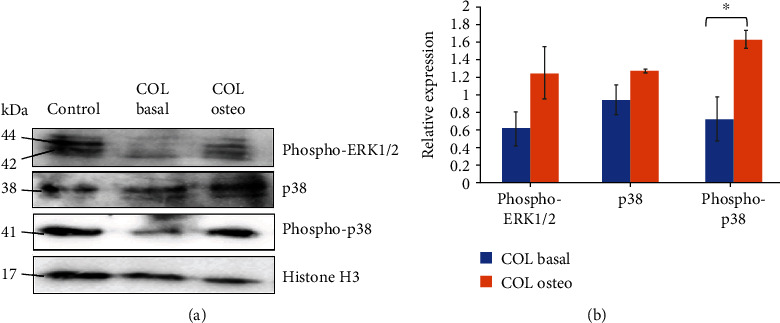
MAPK pathways mediated osteogenesis in WJ-MSCs. Cells were cultured on collagen scaffold at basal or osteogenic condition. (a) Representative Western blot was probed with antibodies as indicated. (b) Relative protein expression level obtained after normalization using histone H3. Data were obtained from three independent biological samples. Asterisk (∗) indicates statistical significance (*p* < 0.05).

Figure 9 shows that the activation of MAPK pathways was slightly suppressed when WJ-MSCs cultured on COL scaffold. However, the p38 pathway was significantly upregulated and reactivation of ERK pathway was also noticeable, demonstrating MAPK pathways have a crucial role in coordinating gene regulatory network during osteogenesis.

## 4. Discussion

Collagen is the most abundantly found protein in ECM. Its versatile properties including high biocompatibility, biodegradability, and easy availability make it a suitable biomaterial to be utilized in tissue engineering as it is the basic structural element in tissues like bones and cartilage [20]. Although collagen had been shown to promote proliferation of BM-MSCs [11, 21], our results showed no obvious proliferative effect on WJ-MSCs. In addition, the expression of genes associated with cell growth such as *FGF2*, *EGF*, and *IGF* was not upregulated in WJ-MSC-COL cultured under basal media. These discrepancies could be attributed to the MSC sources, supplements, and coating methods used in culturing. To avoid growth-promoting effect contributed by fibroblast growth factor (FGF), we omitted this growth factor during cell expansion because FGF was reported to enhance MSC proliferation and differentiation [22, 23].

**Figure 10:**
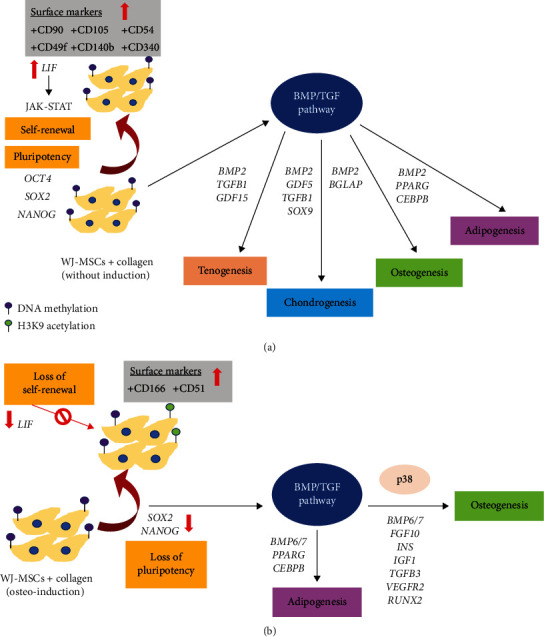
Summary of the underlying mechanisms of collagen modulating lineage differentiation in WJ-MSCs under basal and induced conditions.

As collagen-directed osteogenesis in both basal and induction conditions has been reported in BM-MSCs, we sought to decipher its underlying interplay with WJ-MSCs through gene expression profiles associated with MSC properties as well as the aspect of epigenetic regulatory. We summarized our findings as depicted in Figure 10 and proposed the possible mechanisms. Under the basal condition, COL maintained the pluripotency of WJ-MSCs through hypermethylation at *OCT4* promoter leading to a slight decrease in its expression while other stemness genes were unaltered. Concomitantly, the self-renewal ability of WJ-MSCs was sustained by the upregulation of *LIF*. Based on the altered gene expression profiles, we found that collagen scaffold had also enhanced adhesion, migration, angiogenesis, and directed lineage commitment governed by MAPK signalling as reported previously in other MSCs [24–26]. Upon osteoinduction, the switching of gene regulatory pathways was obvious whereby osteogenic associated partners regardless of the upstream or downstream genes were effectively activated. This was also evidenced by the loss of self-renewal and pluripotency but the increase of histone acetylation marks, suggesting gene transcription associated with osteogenesis was promoted.

As reported previously, we also found that collagen scaffold played roles in adhesion, migration, and angiogenesis as seen by the elevated expression of surface markers such as *CD90*, *CD105*, *CD54*, *CD49f*, *CD140b*, and *CD340*. These cellular behaviours are important during *in vivo* transplantation for ensuring cells are delivered and homed to the damaged sites to perform tissue repair. For example, CD105 (endoglin) is a type 1 transmembrane glycoprotein and coreceptor for the TGF*β* family that plays a crucial role in angiogenesis which is also involved in integrin-mediated adhesion [27]. CD90 (THY-1), a glycosylphosphatidylinositol-anchored glycoprotein, also showed direct interactions with integrins to facilitate cell-cell or cell-ECM interactions [28]. CD140b is a PDGF receptor that improves chemotactic migration to injured tissues and promotes angiogenesis [29]. The study also showed that CD54 (ICAM1) enhanced adhesion and promoted anti-inflammatory effects in MSCs [30]. Besides that, CD49f acted as an inflammation sensor to regulate stem cell behaviours including adhesion, migration, and differentiation in BM-MSCs [31]. The CD51-positive MSCs were found to have the ability in cardiac repair after myocardial infarction in mice [32].

Osteogenesis in the induced WJ-MSCs was accompanied with the downregulation of many surface markers and *LIF* expression, diminishing MSC properties by losing their phenotype in acquire to form osteoblasts. LIF belongs to interleukin-6 family cytokine that activates JAK/STAT3 signalling pathway. It is essential in sustaining pluripotency and self-renewal in embryonic stem cells [33]. LIF and its receptor were found to act as a negative regulator during osteogenesis [34, 35]. The overexpression of LIF was recently reported to promote angiogenesis in BM-MSCs [36]. Furthermore, the suppression of *SOX2* and *NANOG* expression also indicated that WJ-MSCs were differentiated into osteoblastic cells. On the other hand, the overexpression of *OCT4* (*POU5F1*) has been previously found to promote higher osteogenic and adipogenic differentiation in ATM-MSCs, suggesting its dual roles in stemness and differentiation [37].

Mounting evidence has demonstrated that BMP2 has pivotal functions in inducing MSC osteogenic commitment [38, 39]. Interestingly, the *BMP6/7* expression surpassed *BMP2* upon osteogenic induction, suggesting different mechanistic regulations from collagen scaffold alone. The switching role of BMP family members was accompanied by the concurrent upregulation of downstream targets, *VEGFR2* and *TGFB3*, as well as MAPK activation. Several studies reported that BMP2/4 and BMP6/7 attributed to different signalling pathways in promoting osteoblastic differentiation [40]. BMP6 overexpression was found to be more efficient in inducing bone formation in BM-MSCs than BMP2 [41], while BMP7-overexpressing AD-MSCs showed higher osteogenic capacity [42]. In conjunction with other osteogenic factors such as osterix, BMP could induce osteogenesis through both RUNX2 dependent and independent pathways [43]. Besides that, Cabrera-Pérez et al. [44] revealed that BMP2 has enhanced osteogenic differentiation of WJ-MSCs which was found to have poorer osteogenic potential than BM-MSCs. This implies that collagen scaffold could also augment the inferiority of WJ-MSC in osteogenesis by promoting the BMP2 expression. It has been shown that TGFB3 possessed homing effect by recruiting and instructing MSCs to initiate the process of bone regeneration [45]. The combination of TGFB3 with collagen/chitosan sponge enhanced osteogenic differentiation of human periodontal ligament stem cells [46]. Besides that, a significant increase of *VEGFR2* expression upon induction demonstrated it has a role in osteogenic differentiation. VEGFR2 deficiency was found to reduce osteoblast differentiation in osteoprogenitors [47]. In addition, insulin signalling could also be promoted during osteoinduction via accelerating *IGF1* and *INS* expression in WJ-MSCs. Insulin was known to enhance osteoblastic differentiation and bone regeneration in BM-MSCs [48]. IGF1 was found to promote BMP-mediated osteogenesis, and its combination with BMP6 showed a stronger mineralization than BMP2 [49]. It had been shown to be more capable than BMP7 in inducing osteogenesis of BM-MSCs [50]. Furthermore, Feng and Meng [51] demonstrated that IGF1 promoted proliferation and osteogenesis in BM-MSCs via the Wnt/*β*-catenin pathway.

It is known that chondrocyte proliferation and maturation were also mediated through BMP2 during bone development [52, 53]. Not only that, BMP2 was also found to act as a depot-specific regulator of human adipogenesis [54]. Hence, it is difficult to pinpoint a specific lineage commitment attributed by BMP2 regulation in osteo-, chondro-, or adipogenesis because it has crucial roles in these differentiations as reported previously [38, 39, 55]. Nonetheless, this has partially explained the differentiated phenotypes we observed in differentiation assays and the accelerated expression of their upstream or downstream target genes such as *BGLAP*, *GDF5*, *GDF15*, and *TGFB1*. Moreover, potential cross regulation of BMP2/TGF*β*1 and GDF5 in chondrogenesis orchestrated by collagen as the combination of these growth factors had been reported to stimulate chondrogenesis in BM-MSCs [56]. GDF5 has been shown to enhance cartilage cell condensation while inducing chondrogenesis via TGF/Smad and p38 signalling pathways [57, 58]. Through microarray analysis, Tan et al. [59] demonstrated that GDF5 was capable of inducing tenogenesis in BM-MSCs. TGF*β* signalling was also involved in tenocytes recruitment and regeneration at the injury site [60]. Collagen scaffold has been shown to improve tendon and bone healing by increasing the *RUNX2*, *COL1*, and *BMP2* expression in rabbit BM-MSCs [61]. Thus, we postulated that collagen could modulate the convergence of BMP2/TGF*β* and GDF5 pathways in promoting tenogenesis in WJ-MSCs under basal condition, but further investigation is required.

Our results indicated that collagen could promote adipogenesis in WJ-MSCs by increasing the expression of *PPARG* and *CEBPB*. Surprisingly, the upregulation of these two genes was also observed in osteoinduced WJ-MSCs. Despite considerable data showed the antagonistic role of PPARG in osteogenesis, disputes have been reported in BMP-induced osteoblastogenesis [62]. Wang et al. [63] has demonstrated that inhibiting PPARG could suppress osteogenesis induced by BMP2 pathway. Besides that, the PPARG expression has been found in adipocyte and osteoblast, suggesting its roles in fat and bone formation [64]. In addition, posttranslational modifications of PPARG were found to modulate bone formation and resorption [65]. Ectopic expression of CEBPB has been also associated with adipogenesis of hMSC. However, it has been found to work synergistically with PPARG [62]. Although these might explain the unexpected scenario, the proadipogenic effect of dexamethasone (Dex) could not be excluded as it has reported that Dex induced both adipocyte and osteoblast phenotype in BM-MSCs cultured in the same culture media [66]. Della Bella et al. [67] recently demonstrated a remarkable increase in *PPARG* expression in osteogenic induced BM-MSCs but surprisingly the expected upregulation of *RUNX2* was not detected.

Studies showed that epigenetic regulators such as DNA methyltransferases (DNMTs) are essential for maintaining self-renewal in adult stem cells and controlling the expression of pluripotency factor, LIF [68, 69]. Upregulation of *DNMTs* was observed in basal and osteoinduced condition, suggesting possible structural changes in chromatin as collagen and extrinsic factors might provide different cellular cues for cell-ECM interaction in directing fate determination. Generally, the gene expression level is inversely correlated to the methylation status at the promoter region. The increased methylation level at *OCT4* promoter has silenced the gene expression leading to significant impairment of osteogenesis and adipogenesis in BM-MSCs [70]. Li et al. [71] demonstrated that hypomethylation of *ALP* and *RUNX2* was mediated by DNMT3B downregulation when hMSCs cultured in 3D collagen scaffold under oxidative stress. Furthermore, it has been acknowledged that the tendency of MSCs from different origin towards certain cell fate is also dictated by the DNA methylation status. For example, hypomethylation of osteogenic genes was found in BM-MSCs, but in AD-MSCs, adipogenic genes were hypomethylated instead [72]. Yang et al. [73] demonstrated that DNMT1 promoted adipogenesis at early stage but not inhibiting this process at late stage, suggesting DNMT1 governed adipogenic commitment in a time-dependent manner. Besides that, the roles of DNMT3A and DNMT3B in directing osteo-, adipo-, and chondro-lineage specification have also attracted attention. For instance, DNMT3B ablation was shown to impair endochondral ossification and bone remodelling by activating the Notch pathway during fracture repair [74]. DNMT3A and DNMT3B were found to be markedly increased during chondrogenesis in hBM-MSCs via upregulating the expression of chondrogenic marker, *COL2A1* [75].

The regulation of histone acetylation at lysine residues also plays important roles during osteogenesis. Li et al. [76] showed that the upregulation of osteogenic genes was associated with the increased levels of H3K9ac and H3K14ac at the *RUNX2* and *ALP* promoters, while the reduction of these acetylation marks accompanied the decreased expression of *OCT4* and *SOX2*. Besides that, it has been shown that KAT2B deposited H3K9 acetylation marks by recruiting to the BMP promoter thus activating BMP signalling which promoted osteogenic differentiation in AD-MSCs [77]. Knockdown of KAT2B decreased ALP activity and mineralization in osteoblastic cell line [78], highlighting its role in bone formation. These two findings are in agreement with what we observed in osteoinduced WJ-MSC-COL. However, the impact of the remarkable upregulation of *hMOF*, which catalyses H4K16 acetylation, triggered by collagen in basal condition is unclear. We speculate this might be associated with the reprogramming of H4 acetylation in chromatin structure of WJ-MSCs, because hMOF has been found to control self-renewal and pluripotency in ESCs by directly regulating the expression of OCT4, NANOG, and SOX2 [79]. Although histone deacetylation has been associated with osteogenesis [80, 81], we did not see much alteration of *HDAC1* expression but the roles of other HDACs cannot be excluded.

## 5. Conclusions

In conclusion, our results at basal condition indicated that collagen provides a conducive cellular environment for WJ-MSCs by priming cells to respond immediately to differentiation cues. This can be seen by the upregulation of genes involved in BMP/TGF*β* pathway, but limited activation of downstream targets was insufficient to promote vigorous differentiation. However, when WJ-MSCs cultured on collagen scaffold were induced by osteogenic factor, the switching role of the BMP/TGF*β* member and the robust transcriptional activation of downstream targets via p38 MAPK signalling coupled with changes of chromatin landscape was apparent. These findings are useful as the underlying molecular events executed by collagen can help in improving the application of WJ-MSCs in cell therapy.

## Figures and Tables

**Table 1:** List of primer sequences used in RT-PCR.

Gene	Forward primer (5′-3′)	Reverse primer (5′-3′)
*SOX2*	AACCCCAAGATGCACAACTC	CGGGGCCGGTATTTATAATC
*NANOG*	TTCCTTCCTCCATGGATCTG	TCTGCTGGAGGCTGAGGTAT
*CEBPβ*	TTTGTCCAAACCAACCGCAC	GCATCAACTTCGAAACCGGC
*hMOF*	GGCTGGACGAGTGGGTAGACAA	TGGTGATCGCCTCATGCTCCTT
*ACTB*	GTCATTCCAAATATGAGATGCGT	GCTATCACCTCCCCTGTGTG

**Table 2:** Primer sequences for amplifying bisulphite-converted DNA.

Gene	Sequence
*OCT4*	Forward 5′-ATAAAGTGAGATTTTGTTTTAAAAA-3′ Reverse 5′-AACATAAAAAAATCCCCCACAC-3′
*SOX2*	Forward 5′-TGGTAGGTTGGTTTTGGGAG-3′ Reverse 5′-AAACAAATTAATAAACAACCATCCATATAA-3′
*RUNX2*	Forward 5′-GTGGTAGGTAGTTTTATTTTATTTAAGAGT-3′ Reverse 5′-AAAAAACACTCACTAACTCTATTAATCTC-3′

## Data Availability

The data that support the findings of the present study are available upon request.

## Supplementary Materials

Supplementary Materials Supplementary Figure 1: immunophenotyping of the isolated WJ-MSCs.
