# Supplementary figures and images for: Collagen Modulates the Biological Characteristics of WJ-MSCs in Basal and Osteoinduced Conditions
Source: Stem Cells Int. 2022 Aug 28;2022:2116367. doi: 10.1155/2022/2116367 (PMC9441371; doi:10.1155/2022/2116367)

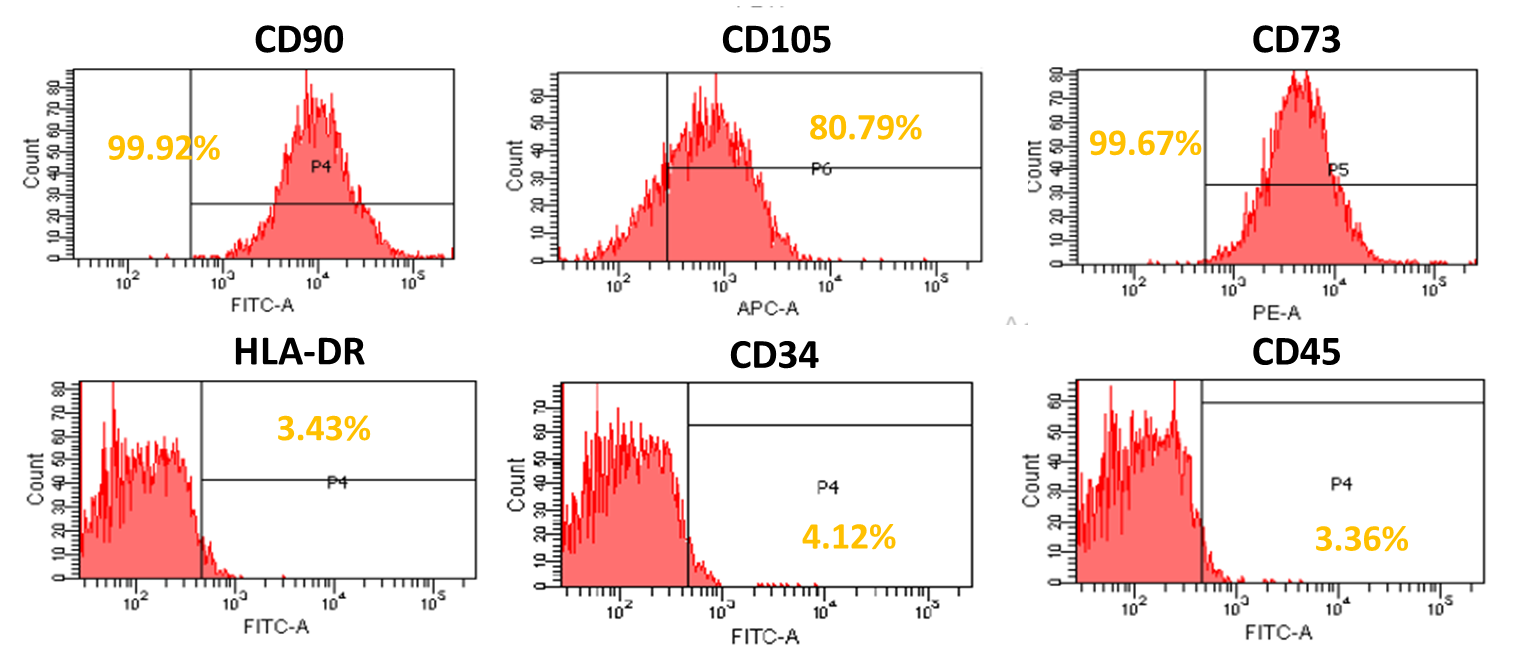


Supplementary Figure 1. Immunophenotyping of the isolated WJ-MSCs.

Supplement: Supplementary Materials — Supplementary Figure 1: immunophenotyping of the isolated WJ-MSCs. [file 2116367.f1.docx]
